# A bibliometric analysis of research on herbal medicine for obesity over the past 20 years

**DOI:** 10.1097/MD.0000000000029240

**Published:** 2022-06-10

**Authors:** Yeonho Seo, Han-Song Park, Hyungsuk Kim, Koh-Woon Kim, Jae-Heung Cho, Won-Seok Chung, Mi-Yeon Song

**Affiliations:** Department of Korean Rehabilitation Medicine, College of Korean Medicine, Kyung Hee University, Seoul, Republic of Korea.

**Keywords:** bibliometric analysis, herbal medicine, obesity, traditional medicine

## Abstract

**Background:**

The aim of this study was to analyze published papers on the use of herbal medicine in obesity research over the past 20 years using bibliometric methods and present an overview of global research trends.

**Methods:**

English articles on herbal medicine for obesity published from 2001 to 2020 were retrieved from the Web of Science Core Collection database using the search terms “herbal” AND “obesity”. Microsoft Office Excel was used to sort and analyze the statistical data. Bibliographic analysis and data visualization were performed using visualization of similarities viewer based on publication year, country of publication, journal, research area, author, affiliated institution, and keywords.

**Results:**

A total of 463 English articles were retrieved, and we observed a trend in which the number of publications on herbal medicine for obesity has gradually increased over the past 20 years. The most productive countries and research organizations in this field were Korea and Kyunghee University, respectively. Many papers have been published in research areas, such as pharmacology pharmacy and integrative complementary medicine, and the journals with the most published articles in this field were Journal of Ethnopharmacology and Evidence-Based Complementary and Alternative Medicine. The main research keywords formed 3 clusters, and keywords with the most occurrences were “obesity,” “adipose-tissue,” and “insulin resistance.”

**Conclusion:**

This study presents an overview of the global research trend of herbal medicine for obesity from the bibliographic analysis. An increased understanding of the recently changing research topics provides a new perspective on future research directions. This study may help guide the research in the field of obesity in the future.

## 1 Introduction

Obesity is a medical condition in which excess body fat accumulates due to an imbalance between energy intake and expenditure.^[1]^ For adults, a body mass index (BMI) of 25.0 to 29.9 kg/m^2^ is defined as overweight, and a BMI of 30 kg/m^2^ or higher is defined as obese.^[2]^ Obese patients are at an increased risk of developing several medical problems, such as hypertension, dyslipidemia, type 2 diabetes mellitus, metabolic syndrome, osteoarthritis, and obstructive sleep apnea.^[3]^ It is widely accepted that obesity is associated with impaired overall health-related quality of life and well-being, including physical and psychosocial functioning, pain experience, and health perception.^[4]^ However, the various factors contributing to obesity, including environmental, psychological, genetic, and physiological causes, make the treatment, management, and prevention of this disease extremely difficult.^[5]^

Research on anti-obesity medications has been actively conducted as a treatment option for obesity. There are several types of Western drugs with different mechanisms of action, including orlistat, lorcaserin, phentermine/topiramate, naltrexone/bupropion, etc.^[6]^ Despite the pharmacological effects of medications, they often have the potential for dangerous adverse effects, such as heart attack and stroke.^[7]^ Therefore, medical professionals and patients seek treatment with herbal medicine with few side effects as an alternative therapy for this medical challenge.^[8]^ As this interest has increased, research on herbal medicines for obesity has been conducted in various aspects, such as drug extraction methods,^[9]^ prescription types,^[10]^ and dialectic methods,^[11]^ as well as treatment effects and adverse effects. Therefore, it is becoming increasingly important to understand scientific research trends in the field of obesity and organize them systematically for more efficient research and development of treatments.

Bibliometric analysis is a quantitative analysis method that uses mathematical and statistical tools to measure the interrelationship and impact of publications within a given research area.^[12]^ Its advantage is that it provides a macro overview of large volumes of academic literature and can be used to efficiently identify influential studies, authors, journals, research organizations, and countries over time.^[13]^ There are international bibliometric studies on obesity, including the following: a publication on global obesity research trends from 1999 to 2017,^[14]^ a report on research trends in global intestinal microbiota studies within the domain of obesity research,^[15]^ and a paper that provided a literature update related to obesity and miRNAs.^[16]^ However, to date, no bibliometric studies have been conducted on herbal medicines for obesity.

The aim of this study was to track the advances in collective knowledge of herbal medicine for obesity treatment using a bibliometric approach and to identify a new perspective on future research directions.

## 2 Materials and methods

### 2.1 Data collection

All data were retrieved from the Web of Science Core Collection database on March 2, 2021, using the following search terms: “herbal” AND “obesity.” Studies published between 2001 and 2020 were included. The Web of Science provides comprehensive publication data and is a widely accepted and frequently used database for the analysis of scientific publications.^[17]^

**Figure 1:**
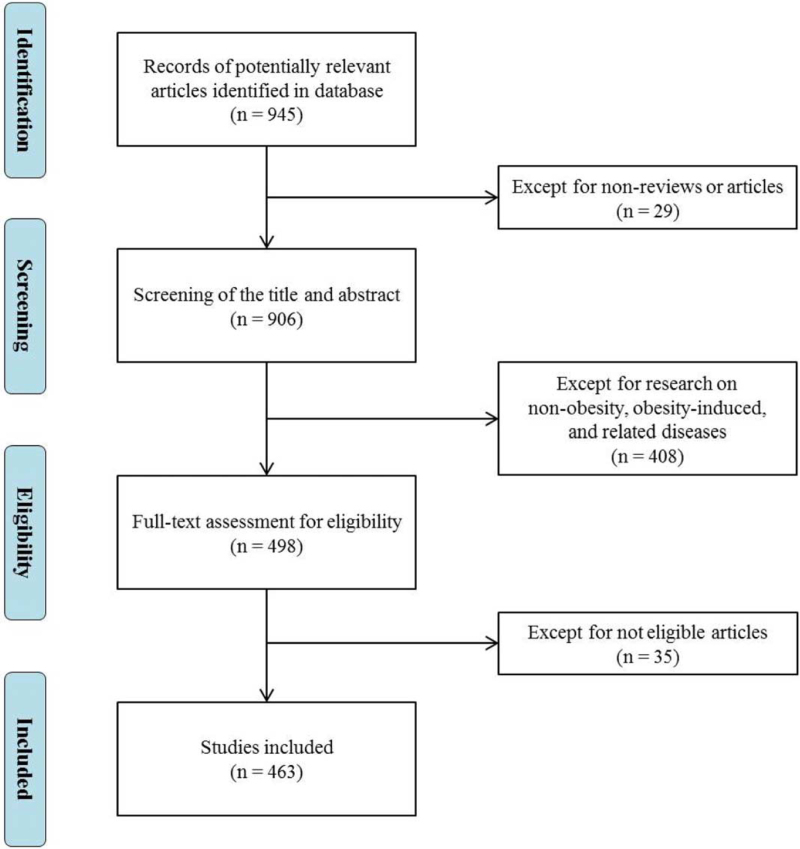
Flow chart of stages of literature search.

The results were restricted to studies published in English, and studies that were not original articles or reviews were excluded (n = 29). Among 906 papers that were obtained, the author read the title and abstract for screening. Inaccurate screening results, which included research on non-obesity and obesity-induced or related diseases (non-alcoholic fatty liver, obesity-related hypertension, polycystic ovary syndrome, etc.) were excluded (n = 408). Another reviewer evaluated the discrepant results by reading the full text of the papers, and those that were not eligible were excluded (n = 35). In this stage, we included the following in the category of herbal medicine: individual herbal medicine or herb-pairs, herbal medicine prescriptions, ingredients of herbal medicines, and natural products. Finally, 463 papers were selected for this study (Fig. 1). Since this study did not include any patient information, the requirement for ethics approval was waived.

### 2.2 Data analysis and visualization

All data were exported from the Web of Science in CSV format for further data processing. Microsoft Office Excel 2016 (Microsoft Corporation, Redmond, WA, USA) was used to stratify and systematically assess the sorted publications. The number of studies was counted by publication year, research area, journal, country of publication, research organization, and author. Then, we calculated the percentage of the total number of articles for each category.

To perform bibliometric analysis and data visualization, all data were imported into visualization of similarities (VOS) viewer v.1.6.11 (Centre for Science and Technology Studies, Leiden University, Leiden, The Netherlands). The VOS mapping method was used to create scientific landscapes and networks, as well as cluster analysis.^[18]^ We generated a density map of published countries and network maps of research organizations, authors, and keywords. The size of the circle nodes in the visualized map showed the number of publications or frequency, the link between nodes showed associations such as collaboration or co-occurrence, and the distance between nodes showed the degree of association. In the cluster analysis of authors and keywords, different node colors represented different clusters, and depending on the similarity threshold between nodes, the number of clusters varied. Clusters were grouped automatically in this study, and the clustering resolution was appropriately adjusted as needed. We aimed to determine the collaboration of authors and co-occurrence of keywords between different clusters as well as articles and to observe the research trends by logging them based on the average publication year and the number of citations. In particular, since keywords that were defined as words used more than 20 times in the titles and abstracts of all papers represent the research topic of the thesis, we calculated the total link strength of a node, which is the sum of the link strengths of this node over all the other nodes.^[19]^ From this analysis, we intended to find popular research topics in herbal medicine research in the field of obesity.

## 3 Results

### 3.1 Analysis of publications by year

**Figure 2:**
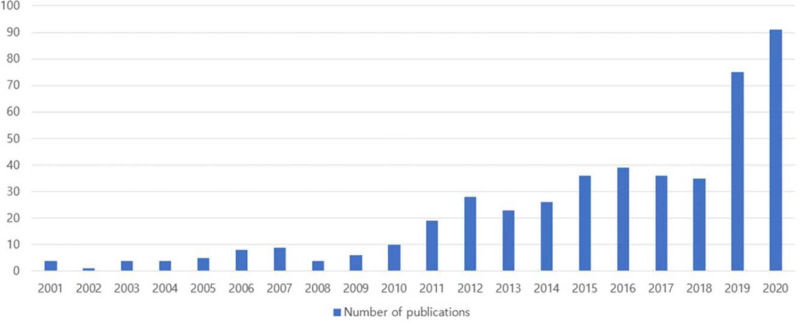
Number of publications by year over the past 20 years.

The number of publications related to herbal medicine for obesity has gradually increased over the past 20 years, with some variations among years. It was shown that the number of papers published in 2019 and 2020 was more than double compared to 2018. In 2020, 91 papers were published, accounting for 19.7% of the total number of articles (Fig. 2).

### 3.2 Analysis of research areas

The top 10 research areas in terms of the number of publications are listed in Table 1. Pharmacology pharmacy (31.32%), integrative complementary medicine (25.49%), and food science technology (12.74%) had the highest number of published papers.

**Table 1:** Distribution by research area.

Ranking	Research area	Records (n)	% (of 463)
1	Pharmacology pharmacy	145	31.32
2	Integrative complementary medicine	118	25.49
3	Food science technology	59	12.74
4	Biochemistry molecular biology	58	12.53
4	Nutrition dietetics	58	12.53
6	plant sciences	51	11.02
7	Chemistry	40	8.64
8	research experimental medicine	31	6.70
9	General internal medicine	23	4.97
10	Science technology other topics	20	4.32

### 3.3 Analysis of journals

When analyzing papers by journal, Journal of Ethnopharmacology (6.48%) and Evidence-Based Complementary and Alternative Medicine (6.48%) published the most papers, followed by BMC Complementary and Alternative Medicine (3.24%), Molecules (2.59%), and Phytotherapy Research (2.16%) (Table 2).

**Table 2:** Distribution by journal.

Ranking	Journal title	Records (n)	% (of 463)	Impact factor^∗^
1	*Journal of Ethnopharmacology*	30	6.48	3.69
1	*Evidence-Based Complementary and Alternative Medicine*	30	6.48	1.81
3	*BMC Complementary and Alternative Medicine*	15	3.24	2.83
4	*Molecules*	12	2.59	3.27
5	*Phytotherapy Research*	10	2.16	4.09
6	*Nutrients*	8	1.73	4.55
6	*Food Function*	8	1.73	4.17
6	*Journal of Medicinal Food*	8	1.73	2.04
6	*Medicine*	8	1.73	1.55
10	*Phytomedicine*	7	1.51	4.27
10	*Scientific Reports*	7	1.51	4.0

∗ The Impact Factor was Reported Based on Journal Citation Reports (JCR) 2019.

### 3.4 Analysis of countries

**Figure 3:**
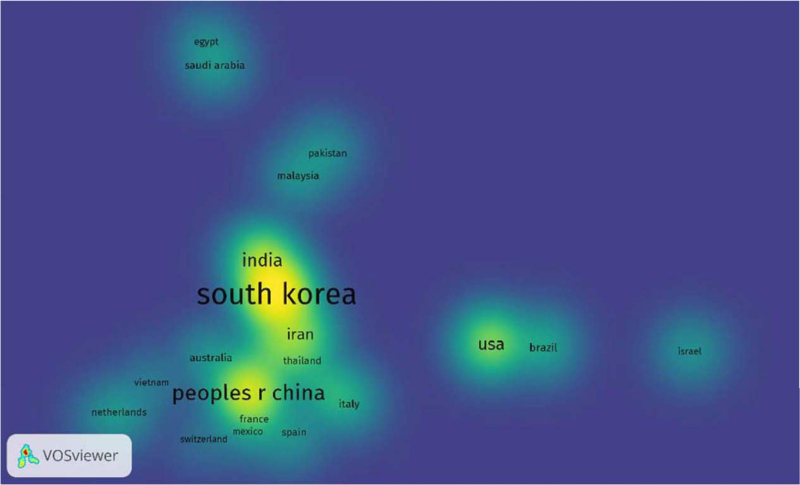
Density map of countries where the papers were published Countries with a greater number of publications have brighter spots.

There were 34 countries that published 3 or more papers. The country of publication was defined as the country to which the main author of the paper belongs. The top 5 countries accounted for over 75% of all published papers globally. South Korea published the most articles (30.13%), followed by China (17.50%), India (11.45%), United States (9.94%), and Iran (7.78%) (Table 3, Fig. 3).

**Table 3:** Distribution by country.

Ranking	Country	Records (n)	% (of 463)
1	South Korea	140	30.13
2	China	81	17.50
3	India	53	11.45
4	United States	46	9.94
5	Iran	36	7.78
6	Japan	28	6.05
7	Brazil	11	2.38
8	Australia	10	2.16
8	Malaysia	10	2.16
8	Saudi Arabia	10	2.16
8	Taiwan	10	2.16
12	Italy	9	1.94
13	France	8	1.73

### 3.5 Analysis of organizations

**Figure 4:**
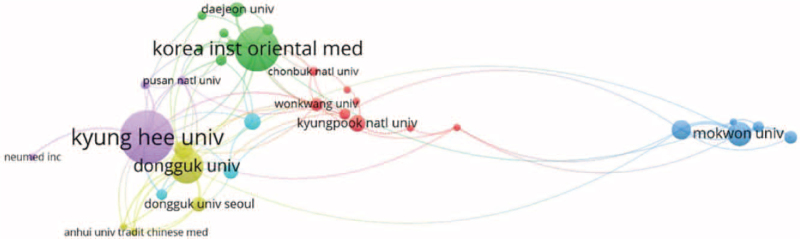
Network map of organizational affiliations.

There were 64 research organizations that published 3 or more papers, and 80% of the top 10 publishing organizations were from Korea. Kyunghee University published the most articles (7.56%), followed by the Korea Institute of Oriental Medicine (7.34%), and Dongguk University (4.54%). A total of 64 organizations were included in the organizational affiliation analysis using VOS viewer, and they were classified into 6 clusters (Table 4, Fig. 4).

**Table 4:** Distribution of organization.

Ranking	Organization	Records (n)	% (of 463)
1	Kyunghee University	35	7.56
2	Korea Institute of Oriental Medicine	34	7.34
3	Dongguk University	21	4.54
4	Mokwon University	13	2.81
5	Gachon University	11	2.38
6	Donge-eui University	10	2.16
7	Mashhad University Medical Science	9	1.94
8	Beijing University of Chinese Medicine	8	1.73
8	Ewha Womans University	8	1.73
8	Kyungpook National University	8	1.73
8	Shanghai University of Traditional Chinese Medicine	8	1.73
12	Tehran University of Medical Science	7	1.51
12	China Academy of Chinese Medical Science	7	1.51
12	Chinese Academy of Science	7	1.51
12	Daejeon University	7	1.51
12	Seoul National University	7	1.51

### 3.6 Analysis of authors

There were 74 authors that published 3 or more papers. Kim Ho-Jun published the most papers with 13 articles (14.35%), followed by Yoon Min-Chung with 12 articles (13.25%) and Bose Shambhunath with 11 articles (12.14%) (Table 5).

**Table 5:** Distribution by author.

Ranking	Author	Records (n)	% (of 463)
1	Kim Ho-Jun	13	14.35
2	Yoon Min-Chung	12	13.25
3	Bose Shambhunath	11	12.14
4	Shun Soon-Shik	9	9.93
5	Ko Seong-Gyu	8	8.83
5	Song Yun-Kyung	8	8.83
7	Seo Chang-Seob	7	7.73
7	Jeong Soo-Jin	7	7.73
7	Shin Hyeun-Kyoo	7	7.73
7	Yoo Sae-Rom	7	7.73
11	Iarijani Bagher	6	6.62
11	Hasani-Ranjbar	6	6.62
13	Choi Myung-Sook	5	5.52
13	Kim Ho-Cheol	5	5.52
13	Kim Min-Young	5	5.52
13	Lenon George Binh	5	5.52
13	Wang Jing-Hua	5	5.52

**Figure 5:**
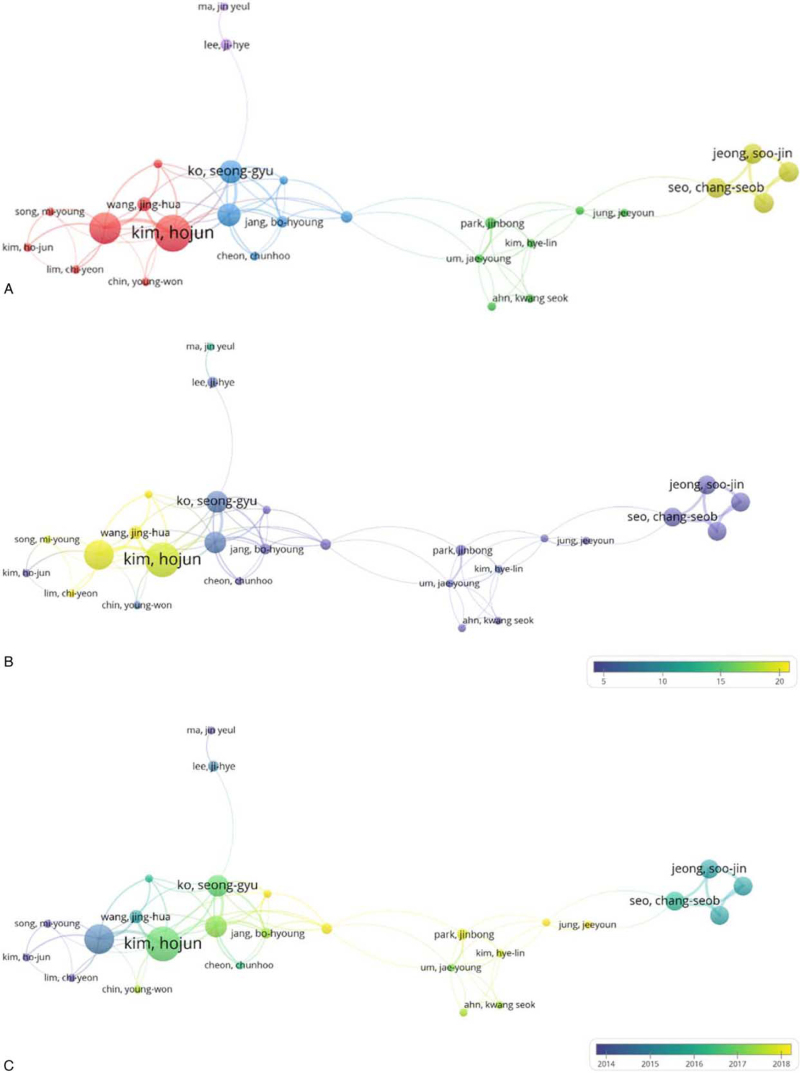
(A) Network map of authors (B) Distribution of authors based on the number of citations (C) Distribution of authors based on the average publication year.

The top 27 authors were classified into 5 clusters: Cluster 1, 8 authors including Kim Ho-Jun, Bose Shambhunath, and Chin Young-Won; Cluster 2, 7 authors including Park Jinbong, Ahn Kwang-Seok, and Jung Jee-Youn; Cluster 3, 6 authors including Cheon Chun-Hoo, Jang Bo-Hyoung, and Kim Hyun-Ju; Cluster 4, 4 authors including Jeong Soo-Jin and Seo Chang-Seob; and Cluster 5, 2 authors who are Lee Ji-Hye and Ma Jin-Yeul (Fig. 5A). The authors were color-coded by VOS viewer based on the number of citations (Fig. 5B) and the average publication year (Fig. 5C). First, when classifying them by the number of citations, the yellow color indicates the authors who published the articles that were cited frequently. Papers written by the authors included in Cluster 1 were cited relatively frequently. Next, when classifying the authors by the average publication year, yellow indicates the authors who published the articles in more recent years. Papers by authors included in Cluster 2 were published relatively recently.

### 3.7 Analysis of keywords

A total of 34 keywords occurred more than 20 times in the titles and abstracts of the 463 published papers included in the present study. Table 6 shows all 34 keywords and their number of occurrences (weight), total link strength, average publication year, and average citations. The top 3 keywords with the most occurrences were “obesity,” “adipose tissue,” and “insulin resistance” (Table 6).

**Table 6:** Keyword classification.

Ranking	Keyword	Weight	TLS	Pub. score	Cit. score	Cluster
1	Obesity	267	622	2015.72	12.54	3
2	Adipose tissue	57	209	2016.51	9.30	1
3	Insulin resistance	65	205	2016.88	11.82	2
4	Extract	62	187	2016.13	8.85	1
5	High-fat diet	55	177	2016.55	11.18	2
6	Adipogenesis	45	157	2016.25	8.44	1
7	Inflammation	48	154	2017.75	9.85	2
8	Expression	40	142	2016.25	9.18	1
9	Ppar-gamma	36	138	2016.14	9.67	1
10	Metabolic syndrome	46	136	2016.02	15.33	2
11	Metabolism	32	119	2016.78	9.17	1
12	Adiponectin	36	118	2016.19	9.12	2
13	Rats	35	105	2015.08	15.58	2
14	herbal Medicine	36	95	2016.06	19.37	3
15	Overweight	31	94	2016.64	8.42	3
16	Oxidative stress	29	91	2017.06	14.19	2
17	Mice	24	84	2015.76	11.03	2
18	Differentiation	25	78	2016.58	7.00	1
19	anti-Obesity	25	76	2017.32	6.84	1
20	Leptin	30	74	2014.60	14.20	2
20	Weight-loss	21	74	2013.67	15.60	3
22	Gut-microbiota	20	72	2018.38	14.14	2
23	Activated protein-Kinase	25	70	2016.80	12.00	1
23	Weight loss	30	70	2013.67	19.83	3
25	Adipocyte differentiation	21	69	2013.81	18.14	1
26	Mechanisms	21	67	2016.40	7.05	2
27	Activation	22	64	2016.41	9.41	1
28	body-Weight	23	63	2013.96	26.13	3
28	in-vitro	25	63	2015.88	16.36	1
30	Glucose	20	61	2016.20	7.90	3
31	Antioxidant	24	55	2015.70	9.92	2
31	Efficacy	22	55	2012.82	18.73	3
33	Green tea	21	47	2015.05	16.33	3
33	Inhibition	20	47	2014.15	12.85	1

Cit.score = average number of citations, Pub.score = average publication year, Ranking = ranked based on the value of TLS, TLS = total link strength, Weight = number of occurrences.

**Figure 6:**
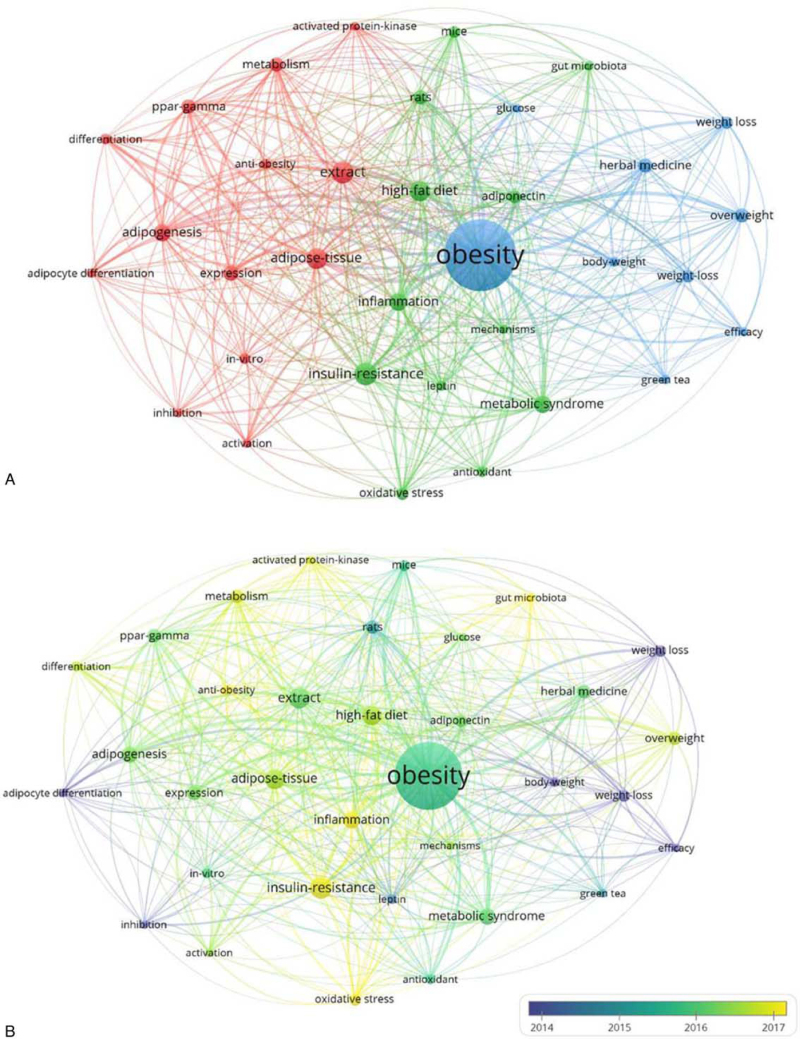
(A) Network map of keywords (B) Distribution of keywords based on the average publication year.

The keywords of the papers were divided into 3 clusters: red, green, and blue (Fig. 6A). We titled each cluster to see popular topics and themes of the research. Cluster 1, colored red, was titled “*in-vitro* experiment with adipose tissue” and included 13 keywords. The strongest keywords in this cluster were “adipocyte,” “adipose tissue,” “adipogenesis,” and “*in vitro*”. Cluster 2, colored green, was titled “*in-vivo* experiment about metabolic system” and included 12 keywords. The strongest keywords in this group were “gut microbiota,” “inflammation,” “mechanisms,” “metabolic syndrome,” “rat,” and “mice.” Cluster 3, colored blue, was titled “an effective way to lose weight” and included nine keywords. The strongest keywords in this cluster were “body weight,” “efficacy,” “herbal medicine,” and “weight loss.”

The keywords were color-coded by VOS viewer based on the publication year, and yellow color indicates that they were published in more recent years (Fig. 6B). The top 3 keywords of recently published articles were “gut-microbiota,” “inflammation,” and “anti-obesity,” whereas the top 3 keywords of the oldest articles were “efficacy,” “weight loss,” and “adipocyte.”

## 4 Discussion

We aimed to perform a bibliometric analysis of herbal medicine research on obesity over the past 20 years to present an overview of global trends and provide a new perspective on future research directions.

We observed that the total number of papers steadily increased during this period. In particular, in 2019 and 2020, 75 papers (16.2%) and 91 papers (19.65%) were published, respectively, which is more than twice as many studies as those that were conducted in 2018, which were 35 papers (7.56%). Recently, as obesity has emerged as a global health issue, many Western medications with strong anti-obesity effects have been developed; however, most of these medications have a risk of severe side effects. For example, sibutramine, a serotonin-norepinephrine reuptake inhibitor, has been reported to frequently cause insomnia, anorexia, constipation, and neurological symptoms.^[20]^ Therefore, the use of natural herbal medicines with fewer side effects and good therapeutic effects can be an alternative treatment for obesity, and it has been shown that it is, in fact, in the spotlight in the literature.

The research areas where the most papers were published were pharmacology pharmacy (145 articles, 31.32%), integrative complementary medicine (118 articles, 25.49%), and food science technology (59 articles, 12.74%). More than half of the studies were conducted in the field of pharmacology and complementary and alternative medicine. Since studies on herbal medicines for obesity have been actively conducted in the field of food engineering, it is expected that herbal medicines prescribed by oriental doctors, as well as obesity-related medicines and supplements using herbal ingredients, are being actively developed.

Based on the journal classification, most papers were published in the Journal of Ethnopharmacology (6.48%) and Evidence-Based Complementary and Alternative Medicine (6.48%). Traditionally, empirical knowledge about the medicinal effects and toxic potential of useful therapeutic agents from plant and animal kingdoms has been passed down. This knowledge has been scientifically proven through observation and experimental investigation on the biological activity of animal and plant substances and has led to the development of valuable drugs, such as morphine and ephedrine. In one of the papers from the Journal of Ethnopharmacology with the herbal extract ALS-L1023 from *Melissa officinalis* L. (Labiatae; lemon balm), which has traditionally been used as a medicinal herb to treat stress, anxiety, insomnia, and weight gain, the researchers showed that it suppressed visceral obesity and insulin resistance in obese female mice by regulating adipogenesis.^[21]^ From this point of view, studies that intend to reveal the therapeutic effects and mechanisms of herbal medicines for obesity could provide scientific evidence and a pharmacological basis for use as complementary drugs for obese patients.

Korea (140 articles, 30.13%) and China (81 articles, 17.5%) are the countries that published the most papers in this field, and many universities and research institutes in these countries are also ranked high when analyzed by research organizations. It appears that East Asia is the leading continent of worldwide research on herbal medicine for obesity, and their results are similar to the results of other bibliometric analysis papers on herbal medicines.^[22,23]^ In particular, research organizations in Korea published almost a quarter of the total number of articles in this study, and this may be due to the fact that various collaborative studies have been conducted at university research institutes where the Department of Oriental Medicine and Food Engineering were jointly established. The subjects of research from these institutions were diverse, and the main subjects were clinical trial studies conducted to determine the efficacy and side effects of herbal medicines. Some studies aimed to determine the effects of existing herbal medicine prescriptions for obesity, such as *Dashiho-Tang*^[24]^ and *Euiiyin-tang.*^[25]^ In addition, studies have shown the effects of self-developed obesity prescriptions, such as *Hanslim*^[26]^ and *Gambisan.*^[27]^ Other studies have investigated the anti-obesity effects of single medicinal ingredients or medicinal roots, such as *Gentiopicroside*^[28]^ and *Puerariae Radix.*^[29]^ In India, the majority of studies were mainly related to Ayurvedic drug, such as research on the anti-obesity effect of *Salacia reticulata* (S. reticulata), which is a herbal medicine used for treatment of early diabetes in Ayurvedic medicine.^[30]^

Authors who have performed extensive research in this field were mostly from the same countries mentioned above. The top 3 authors are Kim Ho-Jun (13 articles), Yoon Min-Chung (12 articles), and Bose Shambhunath (11 articles). Although Kim Ho-Jun and Bose Shambhunath were classified in the same cluster and have conducted joint research, including a study that showed the anti-obesity effect of ephedra extract in obese Korean women,^[31]^ active collaborations were not shown between authors in each cluster as well as top-ranked researchers.

The top 3 keywords with the most occurrences were “obesity,” “adipose tissue,” and “insulin resistance.” This showed that studies on herbal medicine for obesity were conducted with an aim to determine which medicines reduce adipose tissue and control insulin resistance. We classified and clustered the most frequently occurring keywords into specific themes. Cluster 1 was represented by the title “*in-vitro* experiment with adipose tissue” containing terms such as “adipocyte,” “adipose tissue,” “adipogenesis,” and “*in-vitro.*” Excessive adipogenesis, which means abnormal storage of adipose tissue, is one of the major causes of obesity. Therefore, many studies have been conducted to determine whether herbal medicines could be used for the treatment and prevention of obesity by reducing the differentiation of adipocytes based on in vitro experiments at the cellular level. For example, in one study with ancient herbal decoction composed of 4 herbs, *Angelicae Sinensis Radix, Astragali Radix, Jujuba Fructus,* and *Zingiberis Rhizoma Recens,* researchers revealed its anti-obesity function, which consisted in stimulating the browning conversion of white adipocytes in cultured 3T3-L1 cells.^[32]^ Cluster 2 was titled “*in-vivo* experiment about metabolic system,” including keywords such as “gut microbiota,” “inflammation,” “mechanisms,” “metabolic syndrome,” “rate,” and “mice.” In the medical field, animal models are widely used to reproduce human diseases and medical conditions, including obesity. In particular, it has played an important role in studies showing the relationship between gut microbiota composition and the development of obesity, as demonstrated in a bibliometric analysis of research on the role of intestinal microbiota in obesity.^[15]^ A systematic review,^[33]^ including 68 articles that focused on herbal products targeting obesity management through gut microbiota modulation, showed that consumption of herbal products may have beneficial effects on restoring healthy gut microbiome in addition to body fat reduction. Cluster 3 was titled “an effective way to lose weight” and included 9 keywords, namely “body-weight,” “efficacy,” “herbal medicine,” and “weight loss.” This group included clinical studies that supported the effectiveness and efficacy of herbal medicines for obesity. In a systematic review of 12 randomized clinical trials,^[34]^ in 83% of the included trials (10 of 12 randomized controlled trials), BMI or body weight of obese patients reduced significantly. The researchers concluded that this review provided suggestive evidence of the effectiveness of mixed herbal medicines for treating obesity when compared with conventional medicines, placebos, or lifestyle control. In another systematic review in 2021 that included 39 randomized controlled trial,^[35]^ it was reported that herbal medicine could improve body weight and BMI in overweight and obese individuals when used as an adjunct therapy to lifestyle intervention with or without Western medication.

Finally, we analyzed 34 keywords mentioned more than 20 times in the order of the average publication year. The main keywords in relatively recent published papers were “gut-microbiota” (2018.38), “inflammation” (2017.75), and “anti-obesity” (2017.32); alternatively, “efficacy” (2012.82), “body weight” (2013.96), and “weight loss” (2013.67) were found in relatively old papers. From this result, it can be inferred that the trend in recent obesity research is changing from simply finding effective drugs for weight loss to recognizing obesity as inflammation and examining the body's metabolic processes. Therefore, it would be helpful if further rigorous studies are conducted to study the ways in which herbal medicines or natural product complexes affect the intestinal microflora and how they can be used in the treatment of obesity. Additionally, some keywords, such as “caffeine,” “*Citrus aurantium,*” “AMPK (AMP-activated protein kinase),” “Curcumin,” and “ghrelin,” were mentioned less than 20 times, but still deserved our attention.

When looking through all the above keywords and cluster classification, we were able to identify trends in obesity studies that were currently in progress, and we expect that it can be used as a reference to determine the direction of future research. However, this study has several limitations. Only English papers were included in the study. Nevertheless, since English papers accounted for 98.2% of all papers included in our study, the overall results were almost identical to those obtained without language restrictions. Also, the search term “herbal” was selected alone, and similar or higher concepts and words, such as “plant,” “Kampo,” and “decoction” were not added. In bibliometric analysis, the search method is very variable regarding the analysis target based on the search term setting; therefore, setting the search term considering the research purpose and target is very important. In this study, we only used “herbal” as the search term, since this study primarily targets natural products centered on herbal medicine. Additionally, we were unable to classify in detail which drugs or prescriptions were studied and whether they were effective or not, since this paper focused on presenting the overall trend by analyzing “article” and “review” studies together.

## 5 Conclusion

We analyzed 463 papers dealing with herbal medicine in the field of obesity using bibliometric methods. We observed a gradual increase in the number of papers published on this subject, and research in complementary and alternative medicine and pharmacology was active; therefore, research on methods of using herbal medicines for integrative medicine is needed in the future. When analyzing the authors, we expected a more active collaboration between researchers and research institutions considering the diversity of studies. From the keywords analysis, we could see that the trend of obesity research is changing from finding effective drugs for simple weight loss to recognizing obesity as inflammation and examining metabolic processes. If a bibliographic analysis of various oriental medicine treatments, such as acupuncture or moxibustion, is performed in the future, it may help guide the research in the field of obesity.

## Author contributions

**Conceptualization:** Han-Song Park.

**Data curation:** Hyungsuk Kim.

**Formal analysis:** Yeonho Seo.

**Investigation:** Hyungsuk Kim.

**Methodology:** Han-Song Park.

**Software:** Jae-Heung Cho.

**Supervision:** Mi-Yeon Song.

**Validation:** Koh-Woon Kim.

**Visualization:** Jae-Heung Cho.

**Writing – original draft:** Yeonho Seo.

**Writing – review & editing:** Mi-Yeon Song, Won-Seok Chung.
